# The cryoprotective effects of celastrol nanoemulsion on post-thawed attributes and fertilizing ability of cryopreserved buffalo semen

**DOI:** 10.1007/s11259-025-10781-1

**Published:** 2025-06-05

**Authors:** Wael A. Khalil, Salwa A. Elkhamy, Mohamed M. Hegazy, Mahmoud A. E. Hassan, Sameh A. Abdelnour, Mostafa A. El-Harairy

**Affiliations:** 1 Department of Animal Production, Faculty of Agriculture, Mansoura University, Mansoura, 35516 Egypt; 2 Animal Production Research Institute, Agriculture Research Centre, Ministry of Agriculture, Dokki, 12619 Giza Egypt; 3 Department of Animal Production, Faculty of Agriculture, Zagazig University, Zagazig, 44511 Egypt

**Keywords:** Buffaloes, Celastrol nanoemulsion, Semen microbiota, Sperm quality, Oxidative stress

## Abstract

Celastrol (CL), a natural molecule isolated from *Tripterygium wilfordii* Hook. f., possesses notable therapeutic potential across a range of disease states. However, its impact on semen quality following cryopreservation has not been extensively investigated. Thus, this research comprehensively evaluated the cryoprotective effects of celastrol nanoemulsion (CNE) on buffalo semen by analyzing sperm quality, sperm kinematics, acrosome status, oxidative stress, mitochondrial membrane potential, ultrastructure, microbiota, and apoptotic-like changes. Semen samples (n = 45) were collected from five buffalo bulls and subjected to cryopreservation following a standard procedure. Prior to freezing, Tris-extenders were supplemented with celastrol nanoemulsion (CNE) at concentrations of 0 (CNE0), 25 (CNE25), 50 (CNE50), 100 (CNE100), and 200 (CNE200) µg/mL. The results showed that CNE supplementation (excluding 200 µg/mL) significantly improved sperm progressive motility, viability, and membrane integrity in post-thawed samples and after 2 h of incubation at 37 °C with 5% CO_2_. Specifically, sperm kinematic parameters were significantly improved by 50 and 100 µg/mL CNE compared to other treatments (*P* < 0.05). Furthermore, a superior percentage of sperm with intact acrosomes was remarked in the 25, 50, and 100 µg/mL CNE groups than in the other groups (*P* < 0.001). Total antioxidant capacity (TAC) was significantly higher in the CNE100 group compared to the CNE0 group (*P* < 0.001), while no significant differences were found between CNE100 and the other treated groups (*P* > 0.05). Oxidative stress markers (MDA, NO, and H_2_O_2_) were significantly lower in CNE50 and CNE100 groups compared to all other treatments (*P* < 0.001). Furthermore, the CNE 100 µg/mL group exhibited a higher percentage of viable sperm and a lower percentage of apoptotic sperm than other groups (*P* < 0.05). The addition of CNE to the freezing extender significantly reduced the total bacterial count, spore-forming bacteria, and *coliform* bacteria count in post-thawed buffalo bull sperm (*P* < 0.001). Transmission electron microscopy analysis revealed that CNE 100 µg/mL improved and sustained sperm ultrastructure. Notably, the CNE 100 µg/mL treatment also improved the pregnancy rate compared to the control group (82.0% vs. 68.0%). Collectively, the results indicate that CNE at concentrations of 50–100 µg/mL can be effectively used for improving sperm cryo-resistance. This is achieved through improvements in sperm quality and kinematic parameters, attenuation of oxidative stress and microbiota, enhancement of mitochondrial function, preservation of sperm ultrastructure, and improved fertilizing capacity. This study highlights the potential of celastrol nanoemulsion as a nanotechnology-based strategy for optimizing assisted reproductive technologies in livestock.

## Introduction

Water buffaloes are a vital source of animal-derived food products worldwide, providing both milk and meat. However, their reproductive traits present obstacles to global population expansion. Issues such as silent heat, extended gestation periods (El Debaky et al. 2019), and substantial embryonic losses significantly hinder their reproductive performance (Warriach et al. 2015). Artificial insemination offers a promising strategy for improving reproductive efficiency. The application of cryopreserved semen could help overcome these limitations, ensuring the sustainable growth of this valuable species (Warriach et al. 2015). For many years, researchers have worked on perfecting the cryopreservation protocols for buffalo semen (Dessouki et al. 2022; Khalil et al. 2023a). Although the inclusion of herbal extracts or compounds shows potential in reducing oxidative stress during sperm cryopreservation (Khalil et al. 2023a), their poor solubility, bioavailability, and variable effectiveness often impede consistent success (Hari Priya et al. 2024; Khalil et al. 2024b).

Advances in nanotechnology have revolutionized numerous scientific fields, including animal reproduction. The integration of nanomaterials into freezing extenders has demonstrated substantial improvements in post-thaw sperm quality and characteristics (Ismail et al. 2020; Hari Priya et al. 2024; Khalil et al. 2024b). These nanomaterials exhibit enhanced solubility, bioavailability, and efficacy, leading to improved sperm function and plasma membrane integrity, increased sperm cryo-resistance, and protection of sperm DNA (Abdelnour et al. 2023; Nasiri-Foomani et al. 2024a). Furthermore, they enhance the antioxidant defense system within sperm cells and reduce oxidative damage (Abdelnour et al. 2019, 2020). The improved post-thaw characteristics resulting from nanomaterial incorporation have the potential to significantly enhance reproductive efficiency, as evidenced by improved pregnancy rates observed in buffaloes (Shah et al. 2017; Khalil et al. 2023a, b), and rabbits (Dessouki et al. 2024).

Celastrol (CL), a pentacyclic triterpenoid quinone, is a secondary metabolite found in the roots of *Tripterygium regelii* and *T. wilfordii*. This quinone methide triterpene has garnered significant interest for over 70 years due to its promising medicinal properties. The CL exerts its antioxidant effects (Abu Bakar et al. 2020; Qiu et al. 2021) by scavenging reactive oxygen species (ROS) and has been widely studied as a potential anti-cancer agent (Trott et al. 2008). Notably, it holds potential as a therapeutic agent for diabetes-induced testicular injury by mitigating testicular inflammation and attenuating oxidative stress (OS) and apoptosis signaling (Faheem et al. 2024). This molecule exhibits a wide range of pharmacological actions, including antibacterial, anticancer, and anti-inflammatory properties (Abu Bakar et al. 2020). Moreover, CL is a promising therapeutic candidate for intracerebral hemorrhage by supporting OPA1-mediated mitochondrial fusion to counteract OS (Diao et al. 2024). Several studies have suggested that celastrol nanoemulsion (CNE) offers a significant advantage over conventional formulations due to their enhanced stability, solubility, and bioavailability in aqueous environments (Qiu et al. 2021; Li et al. 2024). The CNE have been explored for targeting rheumatoid arthritis through anti-inflammatory and anti-apoptotic actions (Li et al. 2024), exhibiting antimicrobial activity (Padilla-Montaño et al. 2021), and inducing immunogenicity in cancer therapy (Qiu et al. 2021).

Previous in vitro studies have demonstrated that CL possesses diverse biological activities, including enhancing mitochondrial function, exerting anti-inflammatory (Abu Bakar et al. 2020; Faheem et al. 2024), anti-microbial (Padilla-Montaño et al. 2021) and antioxidant effects, and modulating apoptosis. However, the in vivo impact of CNE on buffalo semen quality following cryopreservation, specifically in relation to apoptosis and oxidative stress, remains largely unexplored. Based on these preclinical findings, we hypothesize that CNE offers beneficial in vitro effects by mitigating oxidative stress, supporting sperm function, and maintaining sperm structural integrity throughout the cryopreservation process. To investigate this hypothesis, we conducted a study to evaluate the efficacy of CNE in enhancing post-thawed semen quality in buffaloes. This comprehensive evaluation took a multifaceted approach, examining a wide range of sperm quality parameters including sperm kinematics, oxidative stress, apoptosis, acrosome status, ultrastructure, semen microbiota composition, and mitochondrial membrane potential and their impact on pregnancy rate.

## Materials and methods

### Ethical declaration

 Ethical approval for all practices related to this experiment and animals handling was obtained from the Mansoura University Animal Care and Use Committee (MU-ACUC, AGR.PhD.24.09.1). Additionally, the study adhered to Directive 2010/63/EU and was accompanied in harmony with the ARRIVE guidelines 2.0, ensuring the protection of animals used for scientific purposes.

### Preparation of Celastrol nanoemulsion

Celastrol (ALX-350-332-M025) was purchased from Enzo Biochem Inc. (Long Island, New York, USA). The CNE was prepared using an ultrasonic emulsification method as described by Qiu et al. (2021). Celastrol (1 mg) was initially dissolved in 10 µL of ethanol. This solution was then combined with 25 mg of sesame oil and 25 mg of soybean lecithin. Subsequently, 1 mL of Pluronic F68 solution (100 mg/mL) was gradually added to the drug mixture while stirring for 5 min at room temperature. The mixture was then sonicated in an ice bath for 5 min. Finally, the solution was dialyzed against a 5% glucose solution for 3 h to regulate osmotic pressure and eliminate any residual ethanol. The size and surface charge (zeta potential) of the resulting CNE were characterized using a Malvern ZetaSizer Nano series (Malvern Instruments, Malvern, UK). Transmission electron microscopy (JEOL JEM-2100, Tokyo, Japan) was employed to visualize the morphology of the CNE after negative staining with 2% phosphotungstic acid.

### Animal management and semen collection

The study was performed at Mahalet Mussa Station, Sakha, Kafr El-Sheikh Governorate, Egypt. Healthy adult Egyptian buffalo bulls (*n* = 5, 4–6 years old) were used for this experiment under the International Livestock Management Training Center (ILMTC) guidelines, Kafr El-Sheikh Governorate, Egypt. The bulls were maintained under standardized management conditions at the ILMTC, with routine semen collections performed twice weekly. In this experiment, a total of 45 ejaculates were collected, with nine ejaculates obtained per bull using the artificial vagina technique (maintained at 42–45 °C). Only ejaculates meeting the following criteria were included: sperm motility > 70%, sperm concentration ≥ 8 × 10^8^/mL, and sperm abnormalities ≤ 15% (Abdelnour et al. 2023). Selected ejaculates were pooled and diluted at 37 °C in an extender to a final concentration of 80 × 10^6^ spermatozoa/mL. The animals received a balanced diet to meet their maintenance requirements, as previously explained in (Khalil et al. 2023a).

### Extender formulation and experimental design

The extender utilized for extending buffalo semen was formulated according to the method summarized by Khalil et al. (2023b). Tris 3.03 g, fructose 1.25 g, citric acid 1.68 g, glycerol 6.0 mL, and egg yolk 20 mL, were dissolved in double-distilled water to obtain a final volume of 100 mL. Then, the streptomycin (100 µg/mL) and penicillin (100 IU/mL) were added. The osmolarity (280–300 mOsm) and pH value (6.8–6.9) were adjusted before addition of cryoprotectant. The pooled semen was divided into five aliquots of equal volume according to the study procedure. A standard Tris-based extender was supplemented with various concentrations of celastrol nanoemulsified (CNE), CNE0 (control), 25 (CNE25), 50 (CNE50), 100 (CNE100) and 200 (CNE200) µg/mL. Based on initial sperm concentration, each pool aliquot was diluted with extender supplemented with different concentrations of CNE (0, 25, 50, 100, and 200 µg/mL) to a final concentration of 80 × 10^6^ sperm/mL. The semen was frozen corresponding to the standard formulas manipulated in the ILMTC, as beforehand stated by Khalil et al. (2023a). The frozen semen samples were stored in a liquid nitrogen tank for at least one month.

### Sperm quality attributes

Sperm quality parameters, involving sperm abnormality, progressive motility, viability, and membrane integrity, were evaluated after a 4-hour equilibration at 5 °C. Assessments were conducted immediately post-thawing (37 °C for 30 s) and again after a 2-hour incubation in a 37 °C, 5% CO_2_ incubator (Khalil et al. 2024a). Progressive sperm motility was assessed by a phase-contrast microscope (DM 500, Leica, St. Gallen, Switzerland) provided with a hot stage (37 °C). A ten µL aliquot of diluted semen was placed on a pre-heated slide, covered with a coverslip, and observed at 100x magnification. The same experienced researcher conducted the analysis in a blinded manner, and each sample was evaluated three times.

Sperm viability and morphological characteristics were evaluated using 10 µL aliquots from each sample. The samples were prepared on slides and stained with eosin-nigrosin (Moskovtsev and Librach 2012). A minimum of 200 spermatozoa were examined at 400x magnification using a light microscope. Viable spermatozoa were identified by their unstained or white appearance, whereas non-viable spermatozoa displayed red staining in the head region. Additionally, the rate of recurrence of morphological irregularities in the tail and head regions was recorded.

Sperm membrane integrity was estimated using the hypo-osmotic swelling (HOS) test as previously depicted by Hossain et al. (2010). In brief, five µL of semen was mixed with 45 µL of a hypo-osmotic liquid (75 mOsmol/L) containing sodium citrate and fructose. The blend was incubated at 37 °C for 45 min. Consequently, a drop of the diluted semen was placed on a slide, covered with a coverslip, and examined under a phase-contrast microscope (DM 500, Leica) at 400x magnification. A total of two hundred spermatozoa were counted, and the proportion of spermatozoa exhibiting coiled tails (indicative of intact membranes and a positive HOS response) was determined.

### Computer-assisted sperm analysis

Sperm motion characteristics were estimated using Computer-Aided Sperm Assays (CASA) with Sperm Vision software (Ref: 12,520/5000; Minitube, Tiefenbach, Germany) as described by Dessouki et al. (2022). An Olympus BX microscope (Hamburg, Germany) equipped with a high-speed digital camera (60 frames per second at 60 Hz) was used to capture sperm images under 4x dark-field illumination. In each group, sperm (*n* = 1000–1200) were examined using CASA. The motion features of the sperm were recognized, and several features were observed, including distance curved line (DCL, µm), distance straight line (DSL, µm), distance average path (DAP, µm), velocity average path (VAP, µm/s), velocity curved line (VCL, µm/s), velocity straight line (VSL, µm/sec), linearity (LIN = VSL/VCL), wobble (WOB = VAP/VCL), amplitude of side head dislocation (ALH, µm); straightness (STR = VSL/VAP), and beat cross frequency (BCF, Hz).

### Acrosome status

Acrosome integrity was determined by the Giemsa staining procedure. Briefly, frozen-thawed semen specimens were washed with modified Oliphant and Brackett medium (without albumin) to remove trypan blue (Brackett and Oliphant 1975). After multiple washes, smears were prepared and stained with 10% Giemsa solution for 1 h. Sperm cells were examined under a bright-field microscope to assess acrosome integrity.

- Viable sperm with intact acrosomes: Pink/purple-stained acrosome and white-stained post-acrosomal region.
- Viable spermatozoa with exocytosed acrosomes: White post-acrosomal state, demonstrating true acrosome exocytosis.
- Non-viable sperm with intact acrosomes: Variable staining in the acrosomal region (purple to dark pink) and dark blue/blue post-acrosomal region.
- Non-viable sperm with reacted acrosomes: Lightly stained (white or gray) acrosomal region and a distinctly blue post-acrosomal region, indicating false acrosome reaction.

### Total antioxidant ability and oxidative stress analysis

The frozen-thawed semen samples underwent centrifugation at 4430 × g for 10 min. After centrifugation, the extender was separated from the samples and stored at − 20 °C. The concentrations of total antioxidant capacity (TAC, TA 2513), malondialdehyde (MDA, MD 2529), hydrogen peroxide (H_2_O_2_, HP25) and nitric oxide (NO, NO2533) were evaluated in the post-thawed semen extender at wavelengths of 532, 505, 509, and 540 nm, respectively. The MDA, TAC, H_2_O_2_, and NO demonstrated linearity at concentrations of 100 nmol/mL, 2 mM/L, 1.5 mM/L, and 200 µmol/L, respectively. These parameters have been measured with the spectrophotometer (Spectro UV–Vis Auto, UV-2602; Culver City, CA, USA). The commercial kits were sourced from Biodiagnostic Company (Giza, Egypt) and were used according to the manufacturer’s guidelines.

### Measurement of apoptosis through flow cytometry

Apoptosis-like changes in post-thawed buffalo sperm were assessed using flow cytometry and Annexin V staining, as earlier depicted (Khalil et al. 2023b). Post-thawed semen was centrifuged, and the isolated spermatozoa were resuspended in binding buffer. Subsequently, 100 µL of the sperm suspension was incubated with 5 µL PI (phycoerythrin) and 5 µL Annexin V-FITC (BD Pharmingen™, Denmark) for 15 min in the dark. Following incubation, 200 µL of binding buffer was added. Flow cytometric examination was measured using an Accuri C6 Cytometer (BD Biosciences, San Jose, CA, USA). The percentages of Annexin V-positive (A+), Annexin V-negative (A-), PI-positive (PI+), PI-negative (PI-), and double-positive cells were determined. Sperm populations were categorized into four groups based on the criteria established by Peña et al. (2003). The characterization of viable (A−/PI−), early apoptotic (A+/PI−), apoptotic (A+/PI+) and necrotic sperm (A−/PI +) was done as reported by Khalil et al. (2024a).

### Mitochondrial membrane potential

Post-thawed treated samples were washed with PBS and analyzed for mitochondrial membrane potential (MMP) using a JC-10 assay kit (ab112133; Abcam Company), following the manufacturer’s instructions. Approximately 1 × 10^6^ sperm cells per milliliter were incubated with JC-10 dye in the dark at 37 °C for 1 hour. Subsequently, the sperm cells were washed two times with PBS and examined utilizing flow cytometry (Abdelnour et al. 2022). The percentage of spermatozoa exhibiting orange fluorescence (JC-10 aggregates), indicative of high mitochondrial membrane potential (HMMP), was quantified. 

### Post-thawed microbial counts

Total bacterial counts of the examined semen samples were determined using nutrient agar medium following the method of Maasen and Christensen (1995) and Ngo et al. (2023). Plates were incubated at 37 °C for 24 h prior to enumeration. Aerobic spore-forming bacteria were enumerated by initially heating samples to 80 °C for 10 min in a water bath, followed by cooling to 30–32 °C. These heat-treated samples were then serially diluted and plated on nutrient agar medium, followed by incubation at 30 °C for 48 h. The presence and enumeration of coliform bacteria in the semen samples were also investigated. For this, plates were incubated at 37 °C for 16–18 h before counting (Wright 1984). Six samples were analyzed per group.

### Transmission electron microscopy for assessing sperm ultrastructure changes

Ultrastructural changes in buffalo bull sperm cells were examined using transmission electron microscopy (TEM) following a modified protocol based on Khalil et al. (2019), with some minor modifications. Post-thawed semen samples were fixed in 2% glutaraldehyde in PBS for two hours, then washed three times with PBS via centrifugation (5 min at 4 °C). Specimens were post-fixed in 1% osmium tetroxide for roughly two hours at 4 °C, dehydrated in acetone, and embedded in Epon resin. Ultrathin pieces were prepared using an ultramicrotome, stained with lead citrate and uranyl acetate, and scrutinized by TEM attached with Digital Micrograph Gatan (V 2.11.1404.0) and Soft Imaging Spectator package were employed to complete the image capture. Fields were selected randomly for imaging.

### In vivo fertility

A total of 100 multiparous buffalo cows that exhibited spontaneous estrus were randomly chosen and allotted into two equal groups (50 females per group). The first group served as the control group (CNE0), while the CNE100 group was used for insemination based on the best in vitro results. In vivo fertility was assessed through 56-day non-return rates (NRR). Each buffalo cow underwent artificial insemination using frozen/thawed semen, with all inseminations performed by a single AI expert.

### Statistical analysis

The data was inserted into Microsoft Excel, then transferred into the SPSS program for analysis. First, the normality of the data was checked using the Shapiro-Wilk test. One-way ANOVA (SPSS version 25, USA) was employed to evaluate the impacts of the various CNE treatments on the measured parameters. A *p*-value of less than 0.05 (*P* < 0.05) was considered statistically significant. The following linear model was used: Yij = µ + TRTi + eij, where Yij = observations, µ = overall mean, TRT = effect of the different CNE (i, 1 to 5), and eij = random error. When significant treatment effects were observed, Tukey’s post-hoc test was used for pairwise comparisons between treatment means. The data were visualized using GraphPad Prism 9.0 (GraphPad, USA). Results are presented as Mean ± SE (standard error).

## Results

### Characterization of celastrol nanoemulsion

**Fig. 1:**
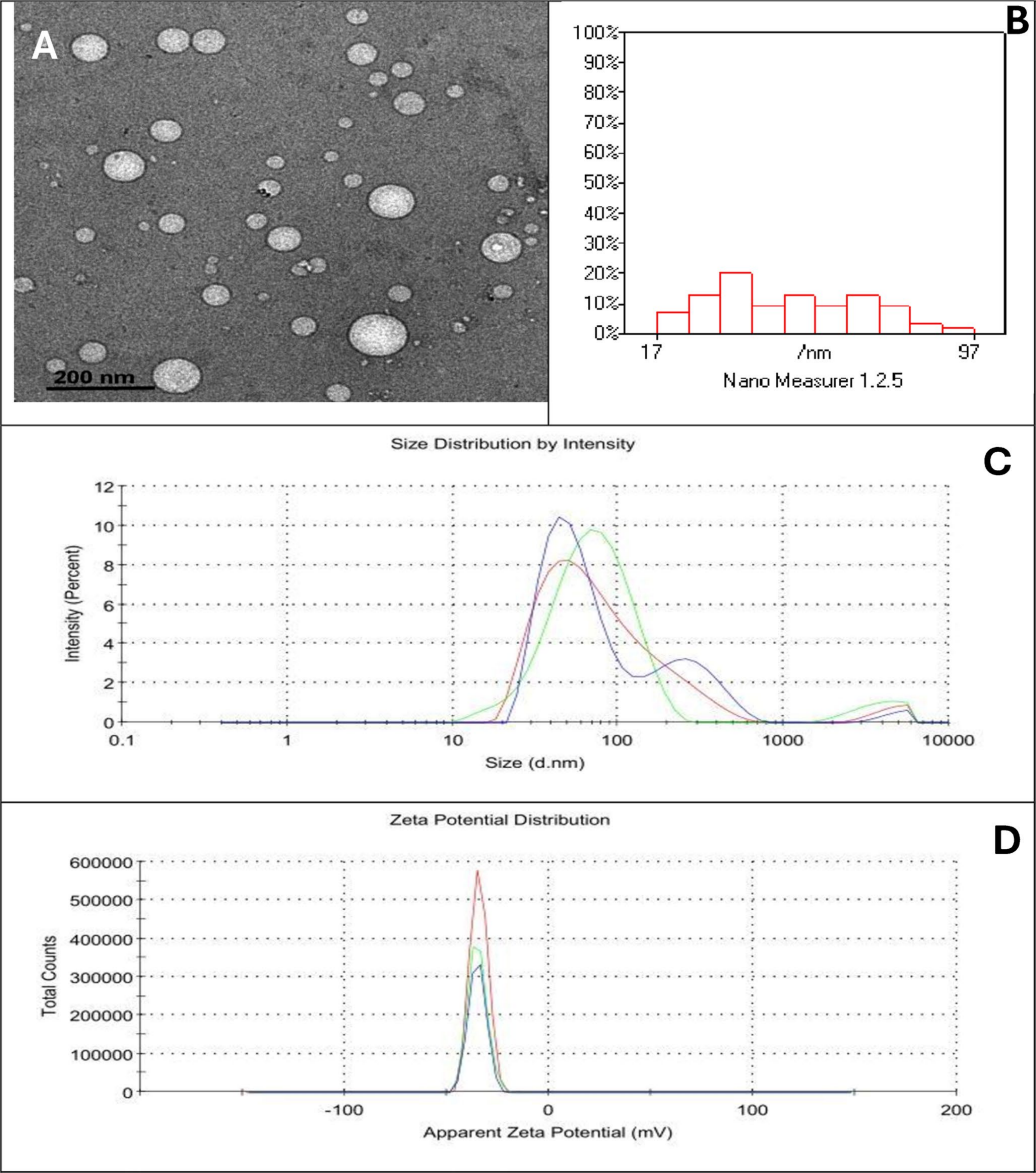
Transmission Electron Microscopy (TEM) images revealed that the celastrol nanoemulsion (CNE) exhibited nearly spherical particles with a uniform size distribution and no observable agglomeration (**A**). The particle size distribution, as analyzed by histogram, demonstrated a narrow peak centered around 17–97 nm, indicating a good distribution (**B**). Zeta size distribution (**C**) by intensity and Zeta potential distribution are also presented (**D**)

The TEM images revealed that the CNE exhibited nearly spherical particles with a uniform size distribution and no observable agglomeration (Fig. 1A). The particle size distribution, as analyzed by histogram, demonstrated a narrow peak centered around 17–97 nm, indicating a good distribution (Fig. 1B). Zeta size distribution (by intensity) and Zeta potential distribution are also presented (Fig. 1C and D). The Z-average size, Polydispersity Index (PDI), and Zeta Potential (ZP) of the CNE were determined to be 63.4 nm, 0.35, and − 35.1 mV, respectively.

### Effects on semen qualities after equilibration (5 °C for 4 h)

Table 1 depicts that the addition of CNE did not affect abnormality, viability, and progressive motility, after equilibration (5 °C for 4 h). However, the addition of CNE improved membrane integrity, with the CNE100 group showing the best values (*P* < 0.05) compared to the control. The addition of CNE at concentrations of 25, 50, or 200 µg/mL did not significantly affect sperm membrane integrity compared to the CNE0 control group (*P* > 0.05). Furthermore, the CNE100 group exhibited greater sperm membrane integrity compared to the control group (*P* < 0.05). There were no significant differences in sperm abnormality across all experimental groups (*P* > 0.05).

**Table 1:** The effects of different celastrol nanoemulsion (CNE 0, 25, 50, 100 and 200 µg/mL) on sperm attributes in buffalo bull semen after equilibration at 5 °C for 4 h (Mean ± SE, *n* = 9)

Extender	Sperm attributes (%)			
	Progressive motility	Viability	Membrane integrity	Abnormality
CNE0	71.7 ± 1.67	73.3 ± 1.86	72.0 ± 2.00^b^	8.0 ± 1.53
CNE25	78.3 ± 1.67	79.3 ± 2.03	80.7 ± 1.45^ab^	6.3 ± 0.88
CNE50	80.0 ± 2.89	82.7 ± 2.96	82.0 ± 2.31^ab^	7.0 ± 1.00
CNE100	81.7 ± 1.67	84.0 ± 1.53	83.3 ± 1.20^a^	6.7 ± 0.88
CNE200	75.0 ± 2.89	77.0 ± 3.46	75.7 ± 3.93^ab^	7.7 ± 1.33
*P value*	0.06	0.07	0.03	0.83

^a−b^ Means denoted with different superscripts in each column are significantly different at *P* < 0.05. Freezing extender supplemented with 0 (CNE0, control), 25 (CNE25), 50 (CNE50), 100 (CNE100) and 200 (CNE200) µg of celastrol nanoemulsion (CNE)/mL

### Effects on post-thaw semen characteristics

Post-thawing, all CNE groups (except CNE200) displayed significant improvement in viability, progressive motility, and membrane integrity related to the CNE-free group (*P* < 0.05, Table 2). Sperm progressive motility was greatest in the CNE50 and CNE100 groups, followed by the CNE25 group, and lowest in the CNE200 and control groups (*P* < 0.05). The viability and membrane integrity of spermatozoa were significantly better in the CNE25, CNE50, and CNE100 groups than in the CNE200 and CNE0 groups (*P* < 0.05). As expected, no statistically significant variations were detected in sperm abnormality rates among the groups (*P* = 0.54).

**Table 2:** The effects of different celastrol nanoemulsion (CNE 0, 25, 50, 100 and 200 µg/mL) on sperm attributes in frozen-thawed buffalo bull semen (Mean ± SE, *n* = 9)

Extender	Sperm characteristics (%)			
	Progressive motility	Viability	Membrane integrity	Abnormality
CNE0	36.7 ± 0.83^c^	39.3 ± 0.75^b^	39.9 ± 1.66^b^	9.6 ± 0.56
CNE25	43.9 ± 1.39^ab^	46.2 ± 0.97^a^	45.8 ± 1.29^a^	9.4 ± 0.58
CNE50	44.4 ± 1.55^a^	46.4 ± 1.66^a^	46.2 ± 1.22^a^	9.3 ± 0.69
CNE100	47.2 ± 0.88^a^	49.7 ± 1.47^a^	48.1 ± 0.92^a^	9.2 ± 0.70
CNE200	38.9 ± 1.39^bc^	40.0 ± 1.12^b^	39.3 ± 1.75^b^	10.7 ± 0.75
*P value*	< 0.0001	< 0.0001	< 0.0001	0.54

^a−c^ Means denoted with different superscripts in each column are significantly different at *P* < 0.05. Freezing extender supplemented with 0 (CNE0, control), 25 (CNE25), 50 (CNE50), 100 (CNE100) and 200 (CNE200) µg of celastrol nanoemulsion (CNE)/mL

### Effects on sperm kinematic parameters

Fortification with CNE at 50–100 µg/mL enhanced DCL (*P* < 0.0001), DAP (*P* < 0.0001), DSL (*P* < 0.0001), VAP (*P* < 0.0001), VCL (*P* < 0.0001), VSL (*P* < 0.0001), STR (*P* < 0.01), and BCF (*P* < 0.0001) compared to other groups (*P* < 0.05, Table 3). LIN (*P* = 0.55) and WOB (*P* = 0.62) remained consistent across all experimental groups. A significantly higher PM was observed in all CNE groups linked to the CNE0 group (*P* < 0.001). The ALH peaked in the CNE100 group, followed by other CNE groups, all demonstrating significant effects. While the CNE200 group showed intermediate STR values relative to the other groups, these differences were not statistically significant (*P* > 0.05).

**Table 3:** The effects of different celastrol nanoemulsion (CNE 0, 25, 50, 100 and 200 µg/mL) on kinematic parameters of frozen-thawed buffalo bull semen (Mean ± SE, *n* = 6)

Item	Treatments					*P* value
	CNE0	CNE25	CNE50	CNE100	CNE200	
PM	37.3 ± 1.48^b^	43.67 ± 1.53^a^	45.67 ± 0.93^a^	48.07 ± 1.12^a^	37.07 ± 0.81^b^	< 0.0001
DAP (µm)	13.7 ± 0.26^b^	14.67 ± 0.44^b^	16.77 ± 0.40^a^	17.67 ± 0.61^a^	14.27 ± 0.19^b^	< 0.0001
DCL (µm)	19.57 ± 0.47^b^	21.57 ± 0.73^b^	24.87 ± 0.49^a^	26.77 ± 1.36^a^	20.57 ± 0.47^b^	< 0.0001
DSL (µm)	10.67 ± 0.09^b^	9.87 ± 0.17^b^	12.17 ± 0.33^a^	12.77 ± 0.42^a^	10.47 ± 0.11^b^	< 0.0001
VAP (µm/sec)	33.87 ± 0.68^b^	32.37 ± 0.76^b^	38.37 ± 1.02^a^	39.27 ± 1.08^a^	31.87 ± 0.34^b^	< 0.0001
VCL (µm/sec)	47.47 ± 0.89^b^	47.57 ± 1.41^b^	56.57 ± 1.35^a^	59.17 ± 2.61^a^	45.77 ± 0.97^b^	< 0.0001
VSL (µm/sec)	21.97 ± 0.21^b^	23.37 ± 0.48^b^	27.87 ± 0.88^a^	28.17 ± 0.61^a^	21.57 ± 7 ± 0.37^b^	< 0.0001
STR (%)	64.87 ± 1.49^b^	67.37 ± 1.43^b^	72.27 ± 0.70^a^	71.57 ± 2.05^a^	67.77 ± 1.31^ab^	0.01
LIN (%)	46.27 ± 0.95	45.77 ± 1.15	48.57 ± 1.12	47.57 ± 1.96	47.27 ± 0.87	0.55
WOB (%)	70.57 ± 1.36	67.57 ± 1.45	67.37 ± 0.99	66.27 ± 1.14	60.07 ± 10.27	0.62
ALH (µm)	1.97 ± 0.09^c^	2.47 ± 0.07^b^	2.57 ± 0.05^b^	3.37 ± 0.09^a^	2.37 ± 0.06^b^	< 0.0001
BCF (Hz)	15.57 ± 0.70^b^	21.07 ± 0.50^b^	22.07 ± 0.56^a^	22.47 ± 0.92^a^	17.07 ± 0.92^b^	< 0.0001

^a−c^ Means denoted with different superscripts in each row are significantly different at *P* < 0.05. DCL, distance curved line (µm); DAP, distance average path (µm); DSL, distance straight line (µm); VCL, velocity curved line (µm/sec); VAP, velocity average path (µm/sec); VSL, velocity straight line (µm/sec); LIN, linearity (VSL/VCL); STR, straightness (VSL/VAP); WOB, wobble (VAP/VCL); BCF, beat cross frequency (Hz) and ALH, amplitude of lateral head displacement (µm). Freezing extender supplemented with 0 (CNE0, control), 25 (CNE25), 50 (CNE50), 100 (CNE100) and 200 (CNE200) µg of celastrol nanoemulsion (CNE)/mL

### Effects on acrosome status

**Fig. 2:**
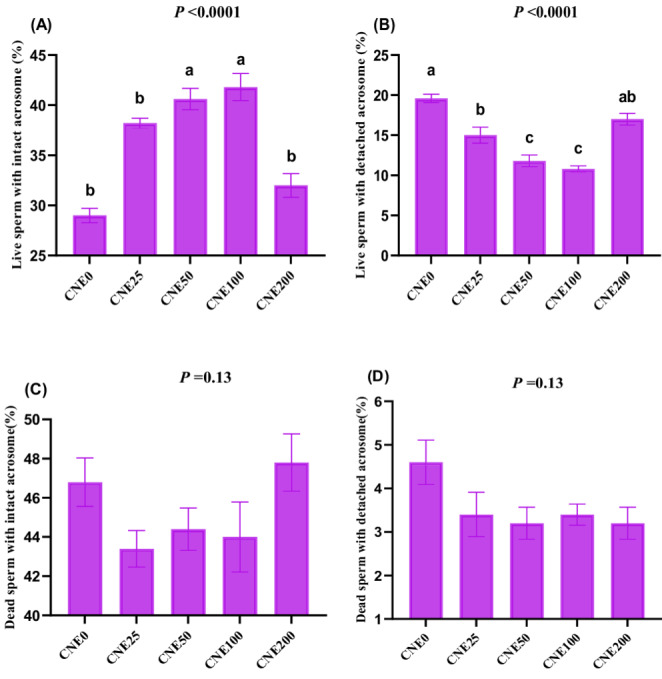
(**A-D**). The effects of different celastrol nanoemulsion (CNE; 0, 25, 50, 100 and 200 µg/mL) on acrosome reaction including live sperm with intact acrosome (**A**), or detached acrosome (**B**), and dead sperm with intact acrosome (**C**) or detached acrosome (**D**) of frozen-thawed buffalo bull semen (Mean ±SE, n=5). ^a-c^Groups with different letters show statistically significant differences at *P* <0.05. Freezing extender supplemented with 0 (CNE0, control), 25 (CNE25), 50 (CNE50), 100 (CNE100) and 200 (CNE200) µg of celastrol nanoemulsion (CNE)/mL

All CNE groups, except CNE200, demonstrated a substantially greater proportion of live sperm with intact acrosomes (Fig. 2A) compared to the CNE0 group (*P* < 0.001). No significant variations were monitored in the proportion of dead sperm with intact (Fig. 2C) or detached (Fig. 2D) across all experimental groups (*P* = 0.13, Fig. 2). The lowest proportion of live sperm with detached acrosomes (Fig. 2B) was observed in the CNE50 and CNE100 groups. The CNE25 significantly increased the proportion of live sperm with detached acrosomes compared to the control group. No statistically significant difference was observed between the control group and CNE200 regarding the proportion of live sperm with detached acrosomes (*P* > 0.05).

### Effects on semen characteristics after incubation at 37 °C and 5% CO_2_ for 2 h

**Fig. 3:**
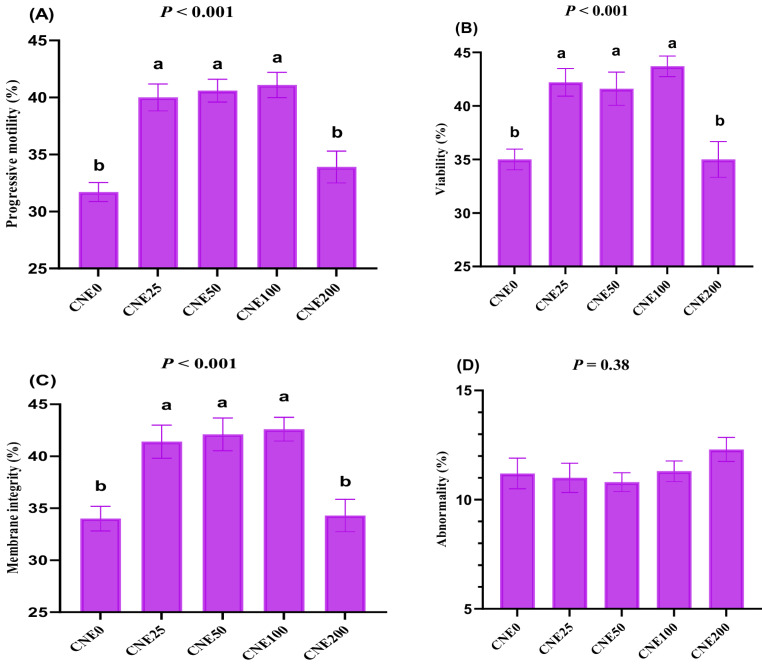
(**A-D**). The effects of different celastrol nanoemulsion concentrations (CNE; 0, 25, 50, 100, and 200 µg/mL) on progressive motility (**A**), viability (**B**), membrane integrity (**C**), and abnormality (**D**) of frozen-thawed buffalo bull semen incubated at 37°C and 5% CO2 for 2 hours (Mean ± SE, n = 9). ^a, b^Groups with different letters show statistically significant differences at *P* <0.05. Freezing extender supplemented with 0 (CNE0, control), 25 (CNE25), 50 (CNE50), 100 (CNE100) and 200 (CNE200) µg of celastrol nanoemulsion (CNE)/mL

Analysis of post-thawed semen variables following a 2-hour incubation at 37 °C and 5% CO_2_ revealed significant enhancements in all CNE treated groups, except for the CNE200 group (Fig. 3). Sperm viability (Fig. 3B), membrane integrity (Fig. 3C), and progressive motility (Fig. 3A) were significantly higher in CNE groups at 25, 50, and 100 µg/mL compared to CNE200 and control groups (*P* < 0.05). Sperm abnormality (Fig. 3D) rates did not differ significantly among all groups (*P* > 0.05).

### Effects on redox status

**Fig. 4:**
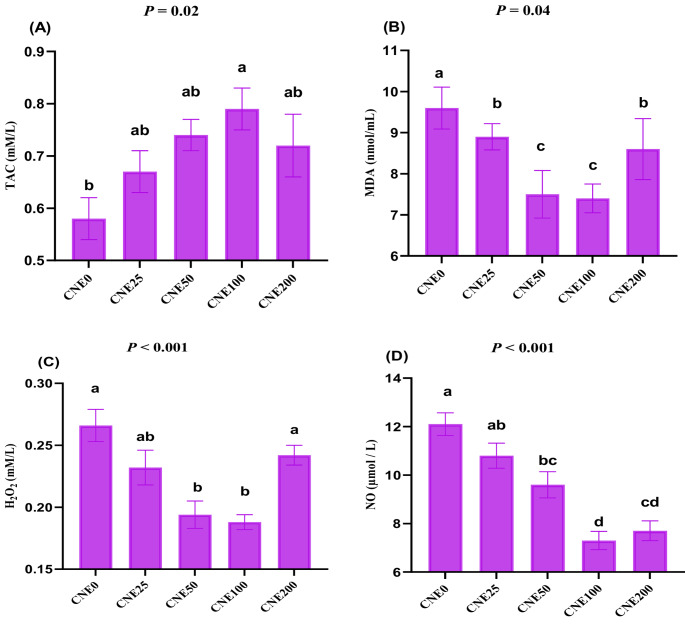
(**A-D**). The effects of different celastrol nanoemulsion (CNE; 0, 25, 50, 100 and 200 µg/mL) on TAC (**A**), MDA (**B**), H_2_O_2_ (**C**) and NO (**D**) in frozen-thawed buffalo bull semen (Mean ±SE, n=5). ^a-d^Groups with different letters show statistically significant differences at p <0.05. Freezing extender supplemented with 0 (CNE0, control), 25 (CNE25), 50 (CNE50), 100 (CNE100) and 200 (CNE200) µg of celastrol nanoemulsion (CNE)/mL

The maximum TAC values were identified in the CNE100 group (Fig. 4A), with no significant differences compared to other CNE groups. All CNE groups (except CNE100) exhibited similar TAC values compared to the control group (*P* > 0.05). All CNE groups displayed lower MDA levels compared to the CNE0 group (Fig. 4B), with the lowest MDA values stated in the CNE100 and CNE50 groups (*P* < 0.05). Groups CNE50 and CNE100 exhibited the lowest H_2_O_2_ levels (Fig. 4C) compared to the control and CNE200 groups (*P* < 0.05), while CNE25 displayed intermediate H_2_O_2_ values. Nitric oxide (NO) levels (Fig. 4D) did not differ significantly between the control group and the CNE25 group (*P* > 0.05).

### Consequences on apoptosis-like changes and mitochondrial membrane potential

**Fig. 5:**
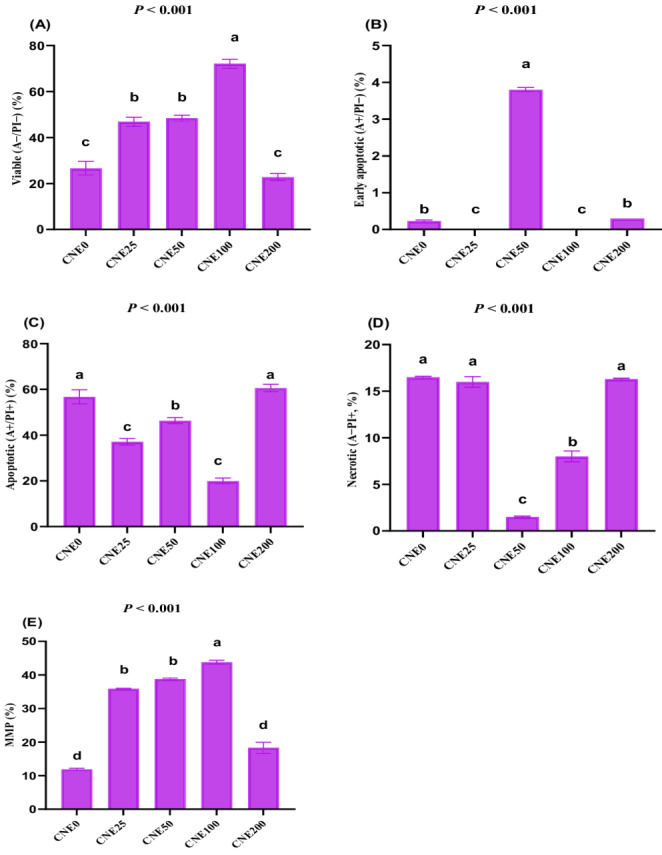
(**A-E**). The effects of different celastrol nanoemulsion concentrations (CNE; 0, 25, 50, 100, and 200 µg/mL) on apoptosis-like changes (viable (**A**), early apoptotic (**B**), apoptotic (**C**), and necrotic (**D**)) and mitochondrial membrane potential (MMP (E)) in frozen-thawed buffalo bull semen were evaluated (Mean ± SE, n = 3). ^a-d^Groups with different letters show statistically significant differences at *P* <0.05. Freezing extender supplemented with 0 (CNE0, control), 25 (CNE25), 50 (CNE50), 100 (CNE100) and 200 (CNE200) µg of celastrol nanoemulsion (CNE)/mL.

Viable sperm counts (Fig. 5A) and MMP (Fig. 5E) were highest in the CNE100 group, followed by the CNE50 and CNE25 groups. The high dose of CNE (200 µg/mL) resulted in similar viable sperm counts compared to the lower doses (*P* > 0.05). Early apoptotic sperm (Fig. 5B) were absent in the CNE100 and CNE25 groups, while the greatest proportion was observed in the CNE50 group (*P* < 0.05). The number of apoptotic sperm (Fig. 5C) was significantly reduced by CNE-added extenders (except for CNE200). The lowest number of apoptotic sperm was observed in the CNE100 group, followed by the CNE25 group (*P* < 0.05). Necrotic sperm counts (Fig. 5D) were lowest in the CNE50 group, followed by the CNE100 group (*P* < 0.05). Notably, all CNE-supplemented groups exhibited better MMP than the control group (free of CNE) (*P* < 0.05).

### Effects on post-thaw semen microbiota

**Fig. 6:**
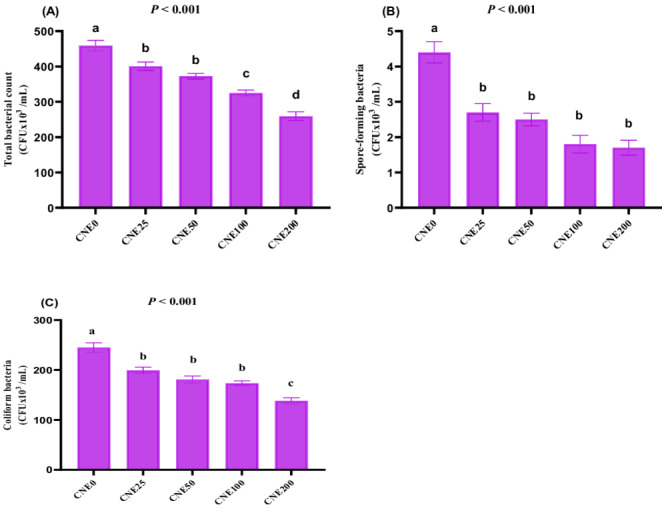
(**A-C**). The effects of different celastrol nanoemulsion (CNE; 0, 25, 50, 100 and 200 µg/mL) on total bacterial count (**A**), spore-forming bacteria (**B**) and coliform bacteria count (**C**) in frozen-thawed buffalo bull semen. Freezing extender supplemented with 0 (CNE0, control), 25 (CNE25), 50 (CNE50), 100 (CNE100) and 200 (CNE200) µg of celastrol nanoemulsion (CNE)/mL. (Mean ±SE, n=6). ^a-d^Groups with different letters show statistically significant differences at *P* <0.05

Regarding the impact of CNE on post-thaw semen microbiota (Fig. 6), it was shown that all CNE levels resulted in a significantly reduced total bacterial count (Fig. 6A), spore-forming bacteria (Fig. 6B), and *coliform* bacteria (Fig. 6C) count compared to the control group (*P* < 0.001). The lowest number of *coliform* bacteria and total bacterial count were recorded at 200 µg/mL of CNE, with significant changes linked to other groups (*P* < 0.05).

### Effects on sperm ultrastructure

**Fig. 7:**
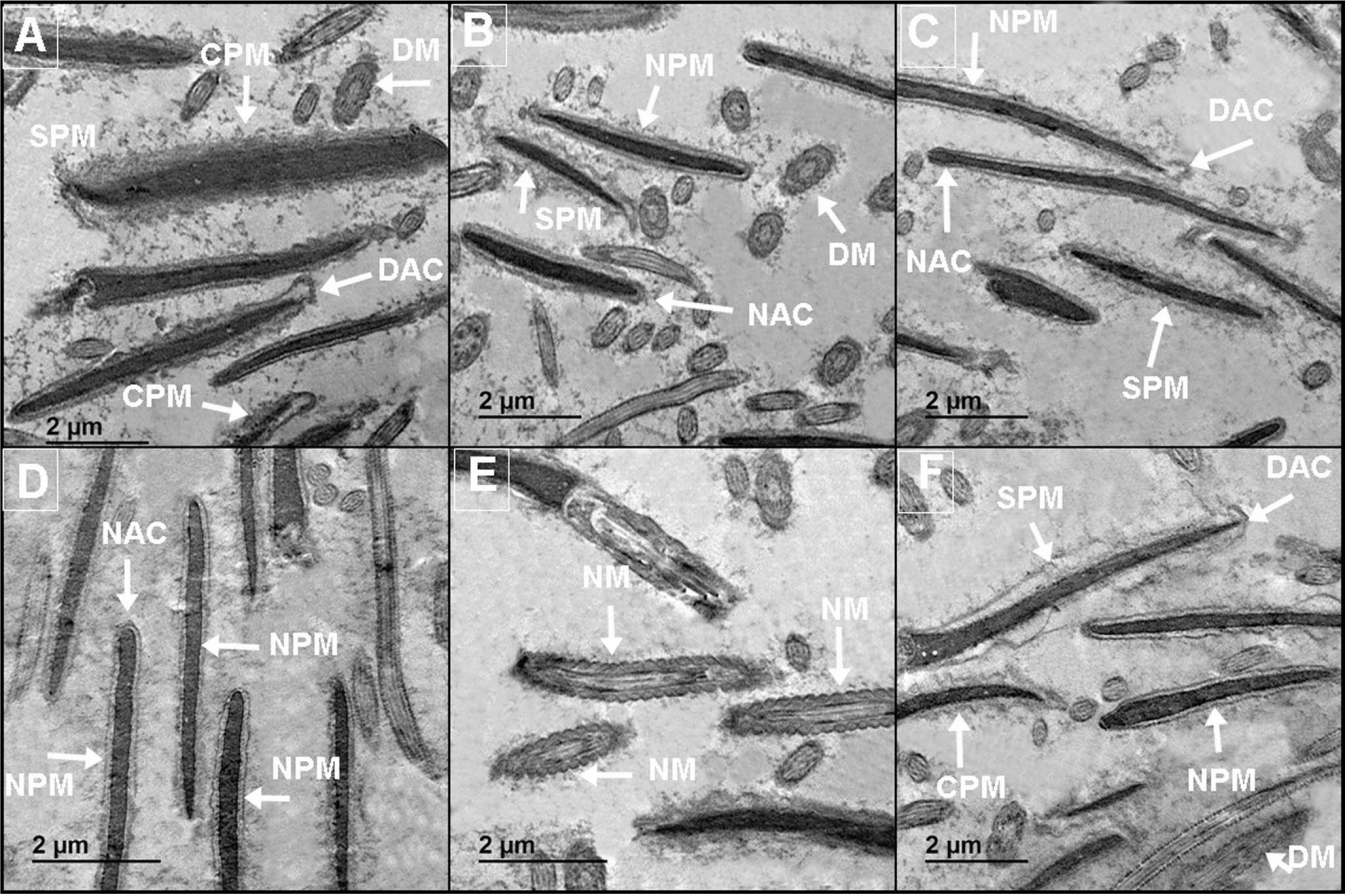
(**A-F**). Impact of varying concentrations of celastrol nanoemulsion (CNE) on the ultrastructure of post-thawed buffalo spermatozoa. In the control group (**A**), sperm cells had mitochondria damage (DM), complete damaged plasma membrane (CPM), damaged acrosomal (DAC). The addition of 25 µg/mL (**B**) or 50 (**C**) µg/mL of CNE exhibited slightly dilated plasma membrane (SDM), slight mitochondria and plasma membrane damages. The freezing extender fortified with100 µg/mL of CNE (**D** and **E**) shown normal mitochondria (NM), intact acrosomal (NAC), and intact plasma membrane (NMP), while the high levels of CNE (200 µg/mL, **F**) had shown slight damages in acrosome region, plasma membrane and mitochondria structure

Figure 7 (A-F) details the effects of increasing CNE concentrations on the ultrastructure of post-thawed buffalo spermatozoa. The control group (Fig. 7A) displayed significant damage, including mitochondrial damage (DM), complete plasma membrane damage (CPM), and damaged acrosomes (DAC). Treatment with 25 µg/mL (Fig. 7B) and 50 µg/mL (Fig. 7C) of CNE offered partial protection, reducing damage to the mitochondria and plasma membrane, though slight dilation of the plasma membrane (SDM) was still observed. The most effective protection was seen at 100 µg/mL CNE (Fig. 7D and Fig. 7E), where spermatozoa exhibited normal mitochondria (NM), intact acrosomes (NAC), and intact plasma membranes (NMP). At the highest concentration tested (200 µg/mL, Fig. 7F), however, slight damage reappeared in the acrosome region, plasma membrane, and mitochondria.

### In vivo fertility

**Fig. 8:**
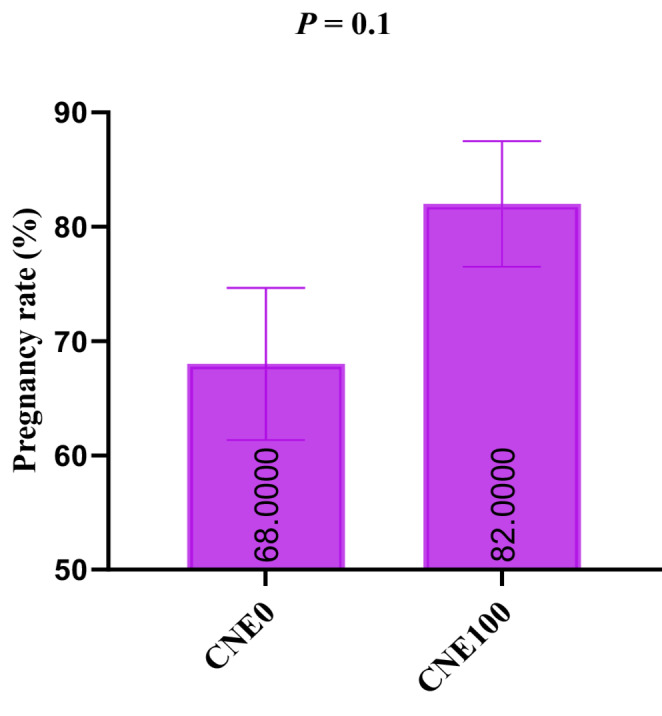
The effects of celastrol nanoemulsion (CNE, 100 µg/mL) on pregnancy rates of buffalo cows

According to the in vitro trials, the best dose was 100 µg/mL of CNE (Fig. 8). Cows inseminated with the addition of CNE (100 µg/mL) had a higher pregnancy rate than the control group (82.0% *vs.* 68.0%).

## Discussion

Sperm cryopreservation has a major contribution to genetic improvements in livestock species. While progress in this technology has been made, it has also been shown to induce oxidative stress, affecting sperm structure and function. Fortifying freezing extenders with phytochemical molecules can mitigate the detrimental effects of cryopreservation processes. However, the limited solubility and availability of these compounds in aqueous solutions have led scientists to improve these features using nanotechnology tools. Nanoemulsions are one such nanotechnological approaches that could enhance solubility and availability, potentially leading to more effective results with lower doses of these nanomaterials. This study aimed to improve the cryopreserved semen of bulls by fortifying extenders with CNE. This study found that adding CNE (25–100 µg/mL) resulted in substantial enhancements in sperm viability, progressive motility, sperm membrane functionality (at post-thawed point and incubation at 37 °C and 5% CO_2_ for 2 h), viable sperm count, sperm kinematic parameters, and acrosome reaction. Supplementation with 100 µg/mL of CNE during cryopreservation notably enhanced TAC and MMP in post-thawed sperm, concurrently decreasing levels of oxidative stress markers (MDA, NO, and H₂O₂) and microbial counts (total bacterial count, spore-forming bacteria, and coliform bacteria count). These results were confirmed by enhancing the pregnancy rates in cows inseminated with semen fortified with CNE (100 µg/mL).

Cryopreservation can significantly reduce sperm motility and viability, as well as damage the sperm plasma membrane due to increased levels of oxidative stress (Abdelnour et al. 2024; Nasiri-Foomani et al. 2024b). This can lead to the premature release of acrosomal enzymes before fertilization, which may decline fertilizing capacity (Asadi et al. 2021). In this study, the addition of CNE (25–100 µg/mL) significantly enhanced sperm motility, viability, and sperm membrane functionality in post-thawed buffalo sperm and after incubation at 37 °C and 5% CO_2_ for 2 h. To counteract the deleterious effects of oxidative stress during cryopreservation, antioxidants are frequently incorporated into semen extenders. However, a significant limitation of many conventional antioxidants is their inability to effectively penetrate the inner mitochondrial membrane. The past few decades have seen considerable progress in the development of novel molecules aimed at targeting mitochondria and mitigating oxidative stress in sperm cells (Khalil et al. 2023b; Li et al. 2024).

Sperm motility is a crucial criterion for successful fertilization, as it requires energy sources for metabolic processes and sperm movement, which is powered by mitochondria. Nanomicelles may across the mitochondria and modulate its activity via interacting with mitochondrial proteins, affecting processes like oxidative phosphorylation (Zhang et al. 2021). These findings align with earlier observations in rabbits (Abdelnour et al. 2020), buffalo (Khalil et al. 2023a, b, 2024b), goats (Ismail et al. 2020), equine (Nasiri-Foomani et al. 2024a).

Enhancing sperm motility by supporting ATP production in mitochondria is an area of interest. Coenzyme Q10 plays a pivotal role in the mitochondrial electron transport chain by facilitating proton and electron transfer (Al-Samarai and Al-Janabi 2021). Complex II functions as a proton pump, while both complexes II and III accept electrons from electron carriers such as FADH2 and NADH, transporting them across the mitochondrial membrane. This cascade of events culminates in increased activity of mitochondrial ATP synthase. Furthermore, it has been reported that CNE can directly interact with ATP synthase, thereby stimulating ATP production in neuron cells (Duberley et al. 2014). Sperm kinematic parameters provide more insight into the types of sperm motility using CASA. Fortification with CNE at 50–100 µg/mL significantly enhanced DAP, DCL, DSL, VAP, VCL, VSL, STR, and BCF compared to other groups. These outcomes were also in line with our previous studies in buffalo (Abdelnour et al. 2023; Khalil et al. 2023a, b, 2024b), implying a substantial enhancement in sperm kinematics due to the addition of several nanoparticles to freezing media.

An intact acrosome is a crucial indicator of a sperm’s ability to successfully fertilize an egg. However, the freezing or chilling process can compromise acrosomal integrity due to high levels of ROS (Abdelnour et al. 2022). Furthermore, the cryopreservation process can induce various alterations within the sperm acrosome and its associated calcium channels (Abdelnour et al. 2019; Hassan et al. 2022). This study demonstrated that CNE significantly enhanced the proportion of live sperm with intact acrosomes while concurrently reducing the incidence of detached acrosomes, particularly within the concentration range of 25–100 µg/mL. Celastrol possesses potent antioxidant capabilities, effectively mitigating lipid peroxidation through direct radical scavenging (Diao et al. 2024; Hong et al. 2023). Moreover, by extending the negative surface charge of the mitochondrial membrane, CL enhances its resilience to the deleterious effects of reactive oxygen species (Diao et al. 2024). Celastrol treatment in obese rats diminished inflammatory reactions via reduced NF-κB action (Abu Bakar et al. 2020). This research demonstrated that CNE significantly improved TAC, while significantly decreasing the levels of MDA, H_2_O_2_, and NO, indicative of robust antioxidant activity that helps maintain cell membrane integrity and the structural integrity of all sperm organelles. These results are consistent with reports in buffalo using various nanomaterials such as propolis-loaded nanoliposomes (Abdelnour et al. 2023), thymoquinone nanoparticles (Khalil et al. 2023b), nanoemulsion essential oils (Khalil et al. 2023a), and metallic nanoparticles (Khalil et al. 2024b).

Another hypothesis suggests that CL may enhance cryoresistance by targeting and modulating specific genes. Celastrol possesses a robust antioxidant capacity by upregulating heat shock genes. Transcriptional profiling uncovered that CNE therapy causes the expression of a battery of antioxidant defense genes in addition to heat shock genes (Klaić et al. 2012). This mechanism is attributed to the ability of CNE to counteract oxidative stress by promoting the synthesis of excess free thiols (Trott et al. 2008). Another study indicates CNE can protect cultured human granulosa-lutein due to its ability to simulate the *SIRT7* gene expression, which contract oxidative stress (Martín-Ramírez et al. 2021).

Celastrol exerts an antioxidative effect on astrocytes by inhibiting the interaction between Nrf2 and Nedd4 genes as well as reduction Nrf2 degradation in ischemic-reperfusion injury (Hong et al. 2023). These findings align with previous studies demonstrating that mitochondria-targeted antioxidants, such as Mito-TEMPO, can protect acrosome integrity and promote MMP during buffalo semen cryopreservation (Kumar et al. 2021). By scavenging excess ROS, which are recycled by the electron transport chain, the antioxidant action of CNE can be replenished. Consequently, CNE can also promote sperm function by supporting MMP, maintaining acrosome integrity, and mitigating ROS and apoptotic sperm.

Cryopreservation can induce apoptosis and mitochondrial damage, leading to sperm dysfunction. However, the addition of CNE promoted MMP, increased the percentage of viable sperm, and decreased the proportion of apoptotic and necrotic spermatozoa in post-thawed buffalo semen. To our knowledge, no previous studies have reported on semen characteristics after treatment with CNE. Mitochondrial function was improved by celastrol administration in obese rats (Abu Bakar et al. 2020). This is due to CL stimulating mitochondrial biogenesis accredited by upregulation of the sirtuin 1 (*SIRT1*) and AMPK signaling pathways. Excessive ROS production triggers oxidative stress, disrupting cellular metabolism and initiating a cascade of pathological events, including increased apoptosis, and oxidative damage leading to infertility (Chianese and Pierantoni 2021). Our conclusions are consistent with the study by Diao et al. (2024) which demonstrated that celastrol effectively mitigates ROS and neuronal apoptosis following intracerebral hemorrhage in rats. Moreover, we previously found that nanoparticles significantly reduced apoptotic sperm and increased sperm viability in buffaloes (Abdelnour et al. 2023; Khalil et al. 2023b, 2024b). However, the cryo-resistance effect and mitochondrial-enhancing properties of celastrol in cryopreserved sperm remain unclear. Similar to our data, Faheem et al. (2024) suggested that CL has the potential to be effective in treating diabetes-induced testicular injury by inhibiting testicular inflammation, apoptosis, and oxidative stress. In contrast, some authors reported the anti-fertility effects of *Tripterygium* glycoside in male (Ge et al. 2023) via upregulating *Cyp19a1* level and decreasing *Pik3ca* and *Pik3cg genes.* Similarly, celastrol’s inhibition of Ca^2+^ currents could be responsible for the antifertility activity of this compound (Bai et al. 2003).

Freezing or chilling processes could induce the activity of pathogenic bacteria, which negatively affects sperm quality. This study showed that adding CNE to freezing media significantly declined the total bacterial count, spore-forming bacteria and *coliform* bacteria count in post-thawed buffalo semen. This reflects the anti-microbial activity of CL as evidenced by El-Deeb et al. (2022) through disrupted the cytoplasmic membrane of bacteria, inhibiting of bacterial protein synthesis, or reducing OS (Al-Janabi and Al-Samarai 2021; Padilla-Montaño et al. 2021). These findings were also consistent with our previous data using metallic nanoparticles (Khalil et al. 2024b). Further advanced techniques are required to support this hypothesis, such as 16 S rRNA gene sequencing.

Transmission electron microscopy (TEM) analysis of sperm ultrastructure provides a novel tool for investigating the mechanisms by which CNE sustains sperm viability and protects sperm cells from the damaging effects of cryopreservation. Cryopreservation displayed significant damage, including DM, complete CPM, and DAC as shown in the control group. Treatment with 25–50 µg/mL of CNE offered partial protection, reducing damage to the mitochondria and plasma membrane, though SDM was still observed. The most effective protection was seen at 100 µg/mL CNE, where spermatozoa exhibited NM, NAC, and NMP. At the highest concentration tested (200 µg/mL), however, slight damage reappeared in the acrosome region, plasma membrane, and mitochondria. These findings align with prior studies demonstrating the efficacy of various nano-phytochemicals in rabbits and buffaloes, including nano-curcumin (Abdelnour et al. 2020), propolis-loaded nanoliposomes (Abdelnour et al. 2023), nano-minerals (Khalil et al. 2024b), nanoemulsion oils (Khalil et al. 2023a) and thymoquinone nanoparticles (Khalil et al. 2023b). Prior research indicates that nanomaterials can mitigate cryo-injury to sperm by creating a protective layer on the sperm surface without disrupting the plasma membrane.

Translating the robust in vitro findings by performing in vivo fertility studies is crucial to confirm these results. Our findings revealed that supplementation with 100 µg/mL of CNE resulted in a significant increase in pregnancy rate in buffalo cows, from 68 to 82%. These results are consistent with earlier reports (Khalil et al. 2023a, b), who found a significant increase in pregnancy rates in buffaloes using nano-material semen administration. This may be related to the ability of CNE to enhance sperm quality by promoting energy supplies, increasing intact acrosome, reducing oxidative stress, semen microbiota and apoptosis.

## Conclusion

Supplementing buffalo semen extender with 50 or 100 μg/mL of celastrol nanoemulsion significantly improved post-thaw sperm quality. This included enhanced sperm motility, viability, acrosome integrity, and kinematic parameters. Furthermore, it mitigated oxidative stress, apoptosis, sustained sperm ultrastructure and reduced the semen microbiota post-cryopreservation. The fertilization rate was improved in cryopreserved sperm diluted in the freezing extender supplemented with 100 μg/mL of celastrol nanoemulsion. Buffalo semen’s functional potential and fertilizing capability are greatly reliant on sperm motility and mitochondrial function. Therefore, focusing on mitochondria-targeted enhancers offers a potentially optimal strategy for maintaining the activity and fertilizing capacity of cryopreserved buffalo semen.

## Data Availability

No datasets were generated or analysed during the current study.

## Abbreviations

ALH: Amplitude of lateral head displacement
BCF: Beat cross frequency
CL: Celastrol
CNE: Celastrol nanoemulsion
CASA: Computer-Aided Sperm Assays
DAP: Distance average path
DCL: Distance curved line
DSL: Distance straight line
HOS: Hypo-osmotic swelling
LIN = VSL/VCL: Linearity
MDA: malondialdehyde
NO: Nitric oxide
OS: Oxidative stress
*SIRT1*: Sirtuin 1
STR = VSL/VAP: Straightness
TAC: Total antioxidant capacity
TEM: Transmission electron microscopy
VAP: Velocity average path
VCL: Velocity curved line
WOB = VAP/VCL: Wobble
